# Validation of the MethylationEPIC BeadChip for fresh-frozen and formalin-fixed paraffin-embedded tumours

**DOI:** 10.1186/s13148-017-0333-7

**Published:** 2017-04-04

**Authors:** Teresia Kling, Anna Wenger, Stephan Beck, Helena Carén

**Affiliations:** 1 Sahlgrenska Cancer Center, Department of Pathology, Institute of Biomedicine, Sahlgrenska Academy, University of Gothenburg, Box 425, 405 30, Gothenburg, Sweden; 2 Department of Cancer Biology, UCL Cancer Institute, University College London, London, UK

**Keywords:** Epigenetics, DNA methylation, 450K, EPIC, Brain tumour, Formalin-fixed paraffin-embedded tissue, DNA restoration

## Abstract

DNA methylation is the most studied epigenetic modification due to its role in regulating gene expression, and its involvement in the pathogenesis of cancer and several diseases upon aberrations in methylation. The method of choice to evaluate genome-wide methylation has been the Illumina HumanMethylation450 BeadChip (450K), but it was recently replaced with the MethylationEPIC BeadChip (EPIC). We therefore sought to validate the EPIC array in comparison to the 450K array for both fresh-frozen (FF) and formalin-fixed paraffin-embedded (FFPE) tumours. We also performed analysis on the EPIC array with paired FF and FFPE samples to adapt to a clinical setting where FFPE is routinely used. Further, we compared two restoration methods, REPLI-g and Infinium, for FFPE-derived DNA on the EPIC array.

The Pearson correlation of β values for common probes on the 450K and EPIC array was high for both FF (mean: 0.992) and FFPE (mean: 0.984) samples. The β values generated from the EPIC array for FFPE samples correlated well with the paired FF tumours, but varied between 0.901 and 0.987. We did note that sample pairs with lower correlation had less bimodal density distributions of β values and displayed higher noise in the copy number alteration plots (generated from the methylation array data) in the FFPE sample. Both REPLI-g and the Infinium restoration for FFPE samples performed well on the EPIC array and generated equivalent correlation scores to the paired FF sample.

## Introduction

DNA methylation is the most studied epigenetic mark, as it is crucial for cell development and regulation of gene expression with aberrations involved in the pathogenesis of several diseases including cancer [1]. DNA methylation is the addition of a methyl group to the carbon-5 position of cytosine, which in mammals primarily takes place on cytosine-phosphate-guanine (CpG) dinucleotides. Several CpG sites clustering in close proximity are termed CpG islands, which are most frequently located in gene promoter regions inferring that unmethylated CpG islands allow for transcription whereas hypermethylated promoter regions repress gene expression [2]. DNA methylation aberrations, e.g. in the form of hypermethylation of the promoter of a tumour-supressing gene, are common in cancer, including paediatric brain tumours, and methylation profiling is an increasingly valuable tool when it comes to tumour classification [3–5]. The Illumina HumanMethylation450 BeadChip (450K), covering 485,000 CpG sites, has been the most common method to assess genome-wide methylation [6], but this array has now been discontinued and replaced with the MethylationEPIC BeadChip (EPIC) which covers 850,000 CpG sites [7].

The use of DNA methylation profiling to classify and subgroup paediatric brain tumours is now moving into clinical diagnostics where the golden standard for histopathological assessments and archiving material is formalin-fixed paraffin-embedded tissue (FFPE). While FFPE is excellent for this purpose, it does induce problems with extraction of nucleotides as the formalin-fixation causes cross-linking and fragmentation of the DNA thus yielding degraded DNA of poor quality [8]. This degradation renders the DNA unsuitable for whole genome amplification (WGA), a vital step in Illumina methylation arrays, thus preventing the use of methylation arrays on FFPE samples. However, two different methods for DNA restoration produced satisfying results for FFPE samples using the 450K arrays [9–11]. To ascertain the transferability to the EPIC arrays, we have compared paediatric brain tumour samples that were analysed with the 450K and the EPIC arrays; both fresh-frozen (FF) and FFPE samples, thereby reporting the first validation of FFPE samples between the platforms. The EPIC array has thus far been validated in comparison with the 450K platform only for cell lines and blood samples [12] and merely one paired sample (renal tumour) of FF and FFPE [7]. We therefore also assessed the accuracy of the EPIC array by comparing nine paired FF and FFPE brain tumour samples and also performed the first comparison on the EPIC array of the two aforementioned protocols for DNA restoration; REPLI-g [9] and Infinium [10].

## Results

**Fig. 1:**
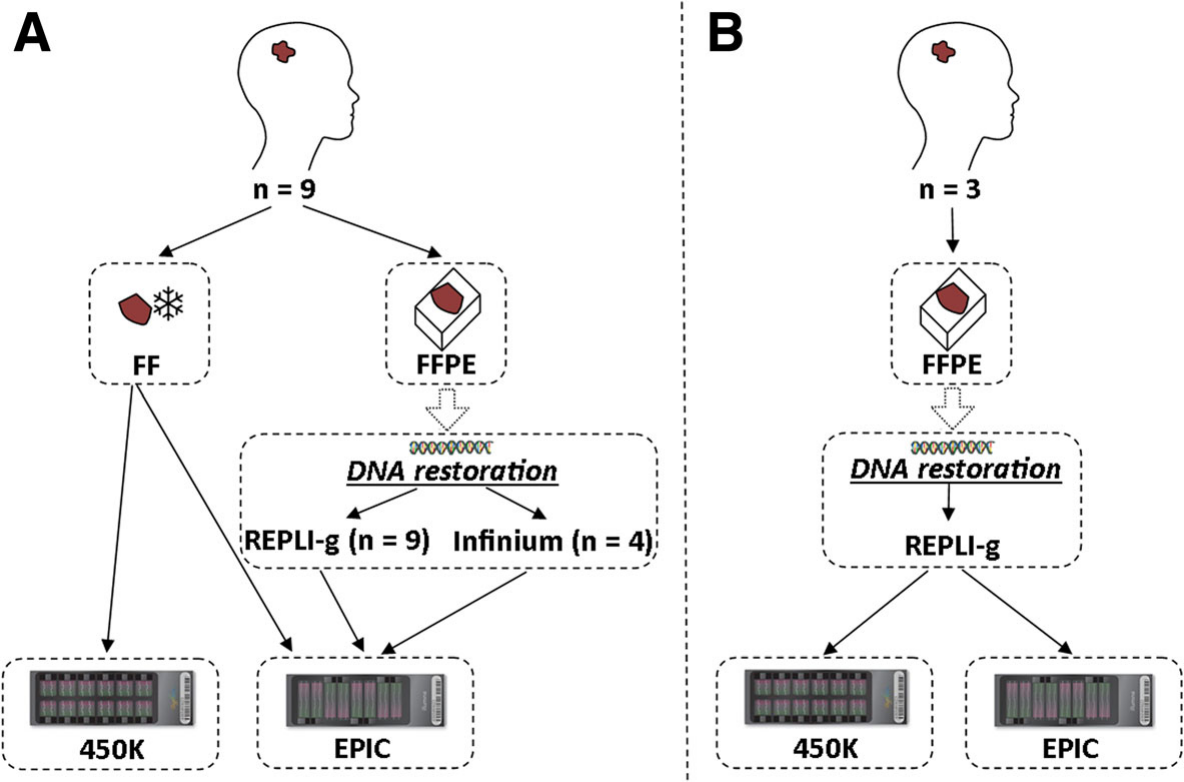
Experimental setup. **a** Nine fresh-frozen (*FF*) paediatric brain tumour samples (*tumours T01-T09*) were processed on the Illumina 450K and EPIC array to compare the platforms. Nine formalin-fixed paraffin-embedded (*FFPE*) samples paired to the FF samples were analysed on the EPIC array to assess the correlation between FF and FFPE and thereby the suitability of the array in a clinical setting (*FFPE samples*). Four of the FFPE samples (*tumours T01*, *T06*, *T08*, *T09*) were restored with both the REPLI-g and Infinium protocol to enable comparison between the methods. **b** Three FFPE samples (*tumours T10-T12*), restored with REPLI-g, were analysed with the 450K as well as the EPIC array to compare the platforms for FFPE samples

This study evaluates the performance of the newly released EPIC platform in comparison to 450K in brain tumour samples. As the analysis of methylation in the clinical setting often is performed on FFPE samples rather than FF, we also evaluated the performance of the EPIC array on nine FFPE samples and compared with the paired FF sample of the same tumour. Further, we assessed two different restoration protocols for the FFPE samples; all nine were restored with REPLI-g, and four of them were also restored with Infinium. The full experimental setup is displayed in Fig. 1. All data is publicly available at GEO; accession number GSE92580.

**Fig. 2:**
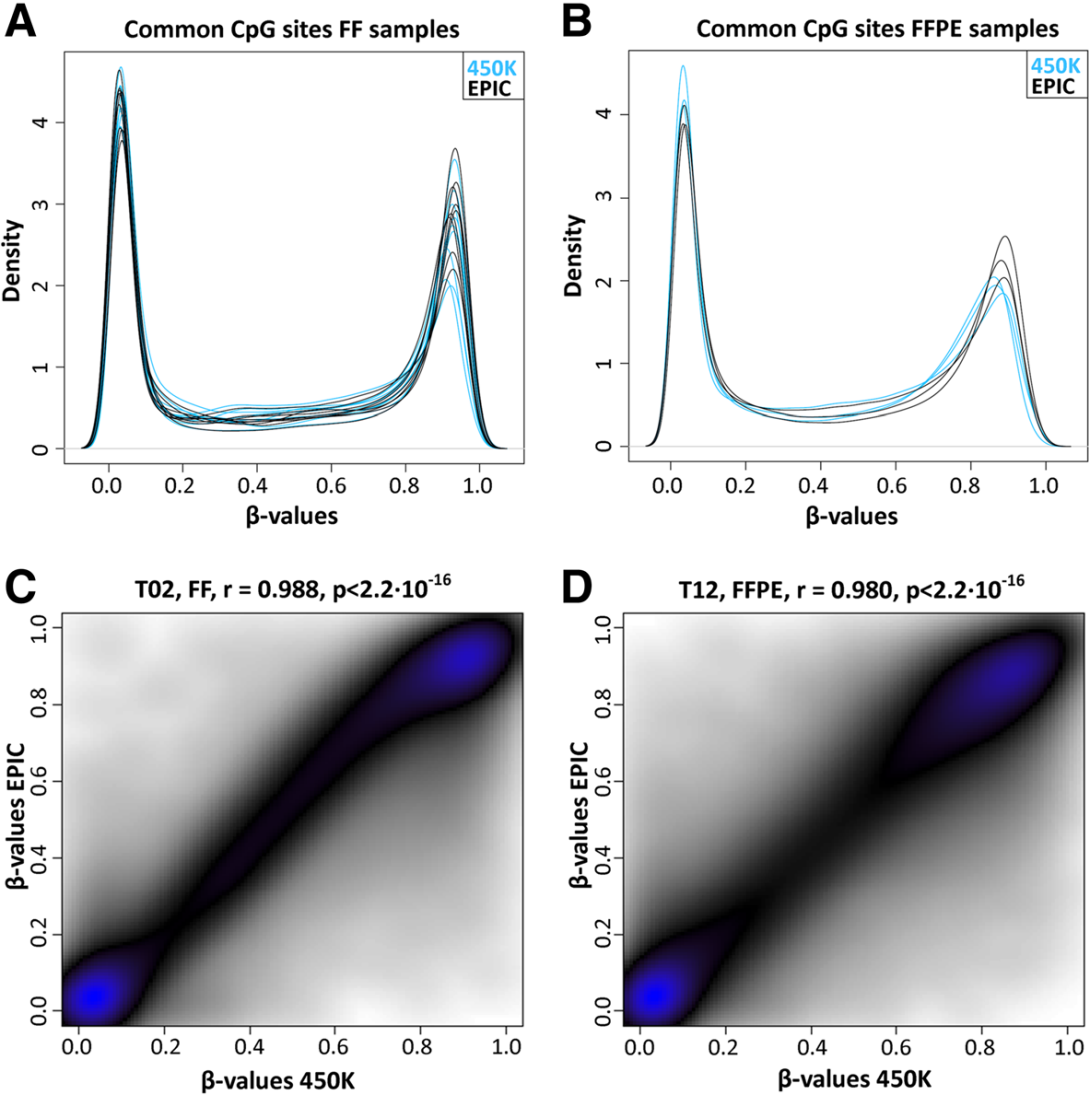
Validation of the EPIC array for fresh-frozen and formalin-fixed paraffin-embedded tumour samples. Density plot of β values for common probes in the 450K and EPIC arrays for **a** fresh-frozen (*FF*) and **b** formalin-fixed paraffin-embedded (*FFPE*) tumour samples. **c** Pearson correlation between obtained β values from the 450K and EPIC arrays for common probes of a FF sample (range: 0.988–0.996, mean: 0.992, std: 0.003, *n* = 9) and **d** FFPE sample (range: 0.980–0.989, mean: 0.984, std: 0.005, *n* = 3)

First, we compared the β values for the ~425,000 CpG sites that are present on both the 450K and the EPIC array for nine FF samples, and three FFPE samples. DNA from the same batch was used for both array types. Density plots of the β values for the common probes (Fig. 2a, b) displayed similar distribution between the arrays for FF and FFPE and good quality for all samples. The Pearson correlation between the 450K and EPIC array ranged between 0.988 (*p* < 2.2 · 10^−16^) and 0.996 (*p* < 2.2 · 10^−16^) for the FF samples (mean correlation: 0.992, std: 0.003, Fig. 2c) and between 0.980 and 0.989 for FFPE (mean correlation: 0.984, std: 0.005, Fig. 2d), confirming previously published results in other tissues that the EPIC array stably reproduces results from the 450K platform also for brain tumours [7, 12].

**Fig. 3:**
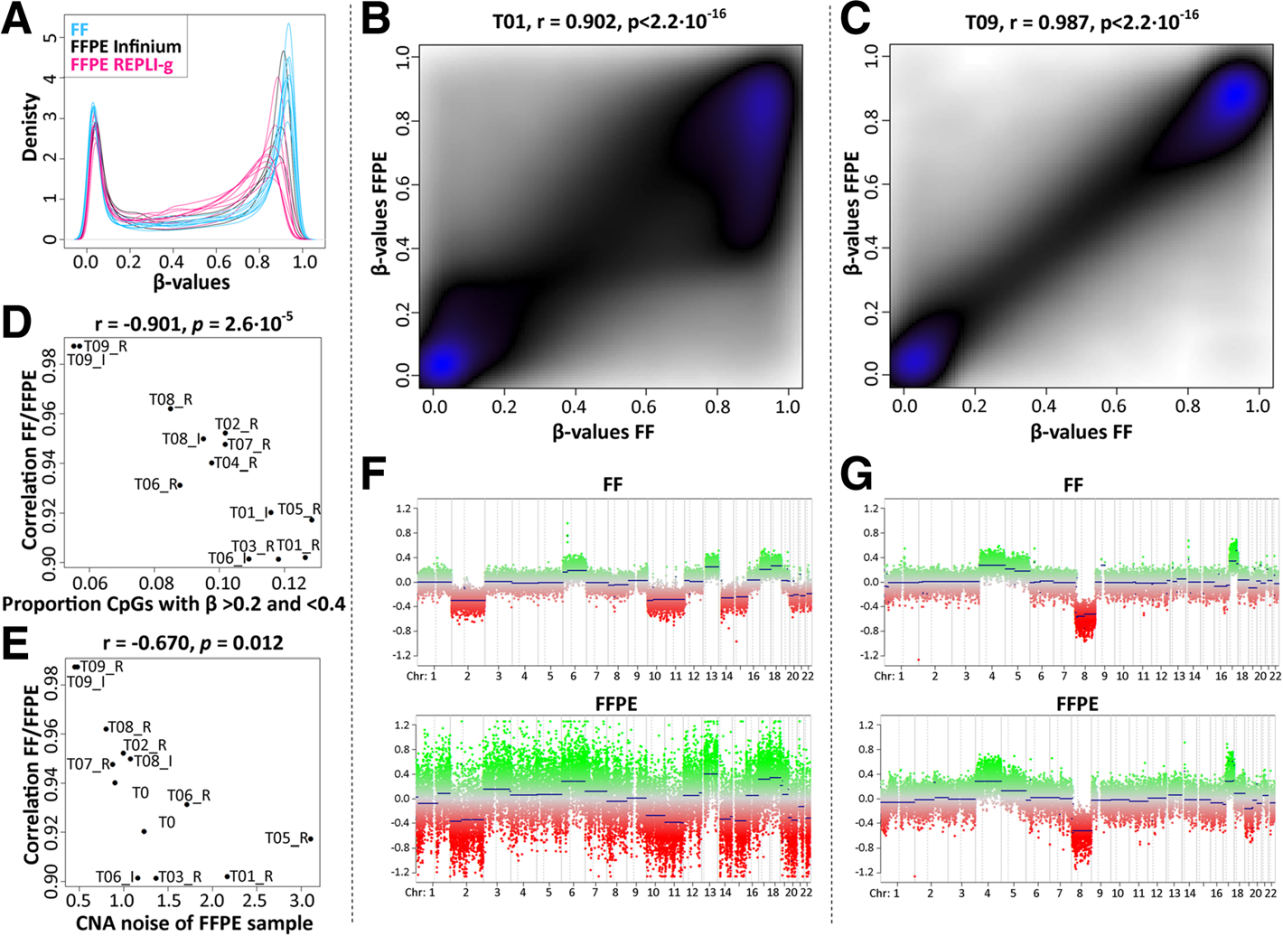
Correlation between paired fresh-frozen and formalin-fixed paraffin-embedded tumour samples on the EPIC array. **a** Density plot of β values from the EPIC array for fresh-frozen (*FF*) and formalin-fixed paraffin-embedded (*FFPE*) samples restored with either Infinium or REPLI-g. Correlation of β values between paired FF and FFPE paediatric brain tumour samples illustrating the sample with **b** the lowest and **c** the highest Pearson correlation respectively. **d** Pearson correlation between paired FF and FFPE samples plotted against the proportion of CpG sites with β values in the low-middle range (between 0.2 and 0.4). **e** Pearson correlation between paired FF and FFPE samples plotted against a noise estimate from FFPE copy number alterations (*CNAs*) indicating a trend of worse correlation with increasing noise. CNA plots for the FF (*upper panel*) and paired FFPE sample (*lower panel*) with **f** the lowest and **g** the highest Pearson correlation respectively.

We proceeded with comparing nine paired FF and FFPE samples on the EPIC array to adapt to a clinical setting where FFPE samples are routinely used. Density plots of the β values displayed some variation, with a tendency for the FFPE samples to indicate worse quality than the FF samples by displaying higher density in the middle-range β values (Fig. 3a). The Pearson correlation between paired FF and FFPE samples ranged from 0.901 to 0.987 (*p* < 2.2 · 10^−16^) for the nine included tumours, with a mean value of 0.938 (std: 0.028, Fig. 3b, c) thus confirming the usability of analysing FFPE samples on the EPIC array. Differences between FF and FFPE samples are expected and well documented in several studies [9, 11, 13] because of the fragmented state and poor quality of FFPE-derived DNA. While the restoration procedures render DNA of sufficient quality for methylation arrays (in particular the WGA step in the process), it does not reach the highest quality of FF-derived DNA. Although some FF-FFPE pairs displayed high correlation in β values, we also observed two tumours with as low correlation as 0.90. The tendency for these samples were similar, indicating that the main effect (Fig. 3b) is that a large portion of CpG sites measured as highly methylated in the FF samples have lost methylation in the FFPE sample. We calculated the ratio between the number of CpG sites with β values in the low-middle range (between 0.2 and 0.4) and the number of CpG sites measured on the array, and we found the ratio to correlate with how well the matched FFPE and FF samples correlated (Fig. 3d). We did not observe any clear correspondence between correlation of FF vs FFPE with neither the age of the FFPE sample nor the quality of the FFPE-derived DNA, measured as delta CT value (compared to QC template) with the Illumina FFPE QC kit (data not shown). Although the age range (14–45 months) of our samples might be too short to notice any clear effects from storage, other studies also report no significant correlation between age of FFPE samples and the performance of methylation arrays [9, 11, 13]. To further compare the FF samples to FFPE, we performed estimation of copy number alterations (CNAs) based on the methylation arrays, using the R package conumee. We did observe a trend of correspondence between noise estimation of the FFPE copy number readouts (as supplied by conumee), and how well the β values for FFPE samples correlated with its matched FF sample (Fig. 3e). The copy number profile of FFPE sample T1 with the lowest correlation to the matched FF sample (0.901) displayed a high noise level (Fig. 3f, lower panel) compared to the corresponding FF CNA profile (Fig. 3f, upper panel). For the samples with high correlation between FF and FFPE, the FF and FFPE CNA profiles were similar (Fig. 3g). This indicated that visual inspection of CNA plots could aid in the assessment of the quality of FFPE samples.

To investigate the distribution of CpG sites that had differing β values in the FF samples compared to the FFPE samples, we specifically analysed all sites with a difference in β value above 0.2 between FF and FFPE samples from the same tumour. For each CpG site, we counted how many of the nine sample pairs that had a β difference >0.2. Very few of the differing sites (268) were present in all nine tumour FF-FFPE pairs, suggesting that CpG sites were affected at random in the FFPE samples making it impossible to predict which sites that are affected when not using paired samples. These 268 differing sites present in all nine samples were spread evenly over the chromosomes.

Finally, we compared two different DNA restoration protocols; REPLI-g and Infinium (see Methods). The main difference between the two restoration protocols is that REPLI-g is performed before bisulfite conversion while the Infinium restoration method is applied after conversion. However, a previous study on the 450K array has compared REPLI-g restoration performed before vs after bisulfite conversion with similar correlation scores to paired FF samples [11]. We compared REPLI-g and Infinium restoration procedures on four FFPE samples before processing on the EPIC array. We observed that the REPLI-g method tends to display higher density in the middle-range β values (Fig. 3a) than the samples processed with the Infinium method, suggesting lower quality in the REPLI-g-restored samples. To further evaluate the performance, we counted the number of failed probes in each sample (Table 1), which were lower in the REPLI-g-restored samples and calculated the correlation to the matched FF sample from the same tumour as summarised in Table 2. Both restoration methods were suitable for restoring FFPE-derived DNA and generating β values similar to the FF samples as observed by the mean correlations; 0.946 (std: 0.037) with REPLI-g and 0.940 (std: 0.037) with Infinium. The correlation to the FF sample was slightly increased using REPLI-g in two of the cases, the Infinium method was performing better in one case, and in one case the correlation to FF was the same. These ambiguous results make it hard to argue for either method, although the REPLI-g has the pro of a less time-consuming protocol and lower cost.

**Table 1:** Number of failed probes (*p* > 0.01) in samples

Sample	FF	FFPE REPLI-g	FFPE Infinium
T01	903	6159	10922
T06	640	1004	6372
T08	874	995	2448
T09	398	316	391

**Table 2:** Comparison of REPLI-g and Infinium DNA restoration of FFPE samples analysed on the EPIC array

Sample	FF vs FFPE REPLI-g (r)	FF vs FFPE Infinium (r)
T01	0.902	0.920
T06	0.931	0.902
T08	0.962	0.950
T09	0.987	0.987
Mean	0.946	0.940

Pearson correlation for β values from four formalin-fixed paraffin-embedded (FFPE) paediatric brain tumour samples, restored with REPLI-g and Infinium, compared with paired fresh-frozen (FF) samples processed on the EPIC array (*p* < 2.2 · 10^−16^ for all sample pairs)

## Conclusions

We have investigated the performance of the new EPIC array compared to the 450K array for brain tumour samples, as well as evaluated the concordance in β values with the EPIC platform between FF and FFPE samples, thus adapting to a clinical setting where FFPE is the standard preservation method of tissue. We also evaluated two different DNA restoration protocols for FFPE samples. To conclude, the reproducibility of 450K data using the EPIC array is good for both FF and FFPE brain tumour samples. The EPIC array, like its predecessor, generates β values from restored FFPE samples similar to those of the paired FF samples. We note, however, that the correlation between paired FF and FFPE samples varies, where noise in the FFPE CNA plot and higher density in the low-mid range (0.2-0.4) region can be an indication of bad quality in the FFPE sample. Further, both evaluated DNA restoration methods yield satisfying results from FFPE samples on the EPIC array with very similar mean correlation scores to the matched FF samples. We can therefore not see any clear benefit for choosing one method over the other as both are suitable for the purpose of restoring FFPE-derived DNA for analysis on the EPIC array.

## Materials and methods

### Samples

Tumour tissue was obtained from children who underwent brain tumour surgery at Sahlgrenska University Hospital 2013–2015 after signed informed consent from the parents. Part of the tumour tissue was preserved as FF in liquid nitrogen and part of it as FFPE. FFPE samples were processed according to standard procedures at the department of Pathology at Sahlgrenska University Hospital by fixation in 4% formaldehyde for 24 or 48 h (depending on the size of the tissue) prior to dehydration with increasing ethanol concentrations and xylene before paraffin infiltration and embedment.

### DNA isolation and quality control

DNA from FF tumours was extracted with the DNeasy Blood & Tissue Kit (69504, Qiagen, Hilden, Germany) according to the manufacturer’s instructions with the addition of lysing the samples with the QIAGEN TissueLyser. FFPE samples (1–3 years old) were sectioned, and DNA was isolated using QIAamp DNA FFPE Tissue Kit (56404, Qiagen) according to manufacturer’s recommendations except de-waxing steps with Xylene was repeated twice, and the tissue was digested overnight. DNA concentration was determined with the Qubit® Fluorometer (Life technologies). The quality of the DNA extracted from FFPE samples was assessed in triplicates with the real-time PCR-based Illumina FFPE QC kit (WG-321-1001, Illumina, Inc., San Diego, CA) according to the provided protocol. FFPE samples with delta CT < 3 compared to the QC template (supplied in kit) was deemed eligible for restoration with REPLI-g or Infinium restoration kit.

### DNA restoration—REPLI-g

DNA from FFPE samples was restored either with (1) REPLI-g prior to bisulfite conversion or (2) with the Infinium restoration kit after bisulfite modification (see below). 1 μg FFPE DNA was used for REPLI-g restoration as described previously [9]. For four out of the nine tumours with paired FF and FFPE, we performed restoration with REPLI-g and Illumina to compare the restoration procedures.

### Bisulfite conversion

500 ng FF DNA, 1 μg REPLI-g-restored FFPE DNA and 1 μg-unrestored FFPE DNA (for restoration with the Infinium kit after bisulfite conversion) was used for bisulfite conversion with the EZ DNA methylation kit (D5001, Zymo Research, Orange, CA) according to the manufacturer’s instructions using the alternative incubation conditions recommended for the Illumina Infinium methylation arrays. Successful conversion was verified by control PCR reactions with a primer set specific for bisulfite-converted DNA, and a primer set for unconverted DNA.

### DNA restoration—Infinium

All of eluate 1 of the bisulfite-converted unrestored FFPE DNA (*n* = 4 samples) was used for restoration with the Infinium HD FFPE DNA Restore Kit (WG-321-1002, Illumina) using the Infinium HD FFPE Restore Protocol supplied by the manufacturer.

### Genome-wide methylation arrays

Infinium HumanMethylation450 BeadChips and Infinium MethylationEPIC BeadChips (Illumina) were used for the determination of methylation levels of more than 450,000 and 850,000 CpG sites, respectively, as previously described [14]. 4 μl of eluate 1 of the FF-, REPLI-g-restored and Infinium-restored bisulfite-converted DNA was used for the methylation arrays according to Illumina’s protocols. Unrestored FFPE DNA was not processed on the methylation arrays.

### Data analysis

Raw methylation data was normalised using Noob-normalisation with the R minfi package [15–17]. CpG sites with detection *p* value >0.01 were regarded as failed and were assigned as missing. Also, 43,254 CpG sites with probes identified to be cross-hybridizing according to Pidsley et al. [12] were removed prior to analysis. For the comparisons between the 450K and EPIC arrays, we analysed probes present on both platforms. CNA analysis was performed using the conumee R package [18]. As reference samples for the CNA analysis, we used EPIC array data from three samples of non-malignant tissue-associated fibroblasts (NAF), and five samples of infant blood from archival Guthrie cards available in the public GEO dataset: GSE86831.

## Abbreviations

450K: Illumina HumanMethylation450 BeadChip
CNA: Copy number alteration
CpG: Cytosine-phosphate-guanine
EPIC: MethylationEPIC BeadChip
FF: Fresh-frozen
FFPE: Formalin-fixed paraffin-embedded
NAF: Non-malignant tissue associated fibroblasts
WGA: Whole genome amplification

## References

[1] Jones PA, Baylin SB (2007). The epigenomics of cancer. Cell.

[2] Herman JG, Baylin SB (2003). Gene silencing in cancer in association with promoter hypermethylation. N Engl J Med.

[3] Sturm D, Witt H, Hovestadt V, Khuong-Quang DA, Jones DT, Konermann C, Pfaff E, Tonjes M, Sill M, Bender S (2012). Hotspot mutations in H3F3A and IDH1 define distinct epigenetic and biological subgroups of glioblastoma. Cancer Cell.

[4] Hovestadt V, Remke M, Kool M, Pietsch T, Northcott PA, Fischer R, Cavalli FM, Ramaswamy V, Zapatka M, Reifenberger G (2013). Robust molecular subgrouping and copy-number profiling of medulloblastoma from small amounts of archival tumour material using high-density DNA methylation arrays. Acta Neuropathol.

[5] Danielsson A, Nemes S, Tisell M, Lannering B, Nordborg C, Sabel M, Carén H (2015). MethPed: a DNA methylation classifier tool for the identification of pediatric brain tumor subtypes. Clin Epigenetics.

[6] Sandoval J, Heyn H, Moran S, Serra-Musach J, Pujana MA, Bibikova M, Esteller M (2011). Validation of a DNA methylation microarray for 450,000 CpG sites in the human genome. Epigenetics.

[7] Moran S, Arribas C, Esteller M (2016). Validation of a DNA methylation microarray for 850,000 CpG sites of the human genome enriched in enhancer sequences. Epigenomics.

[8] Gilbert MT, Haselkorn T, Bunce M, Sanchez JJ, Lucas SB, Jewell LD, Van Marck E, Worobey M (2007). The isolation of nucleic acids from fixed, paraffin-embedded tissues-which methods are useful when?. Plos One.

[9] Thirlwell C, Eymard M, Feber A, Teschendorff A, Pearce K, Lechner M, Widschwendter M, Beck S (2010). Genome-wide DNA methylation analysis of archival formalin-fixed paraffin-embedded tissue using the Illumina Infinium HumanMethylation27 BeadChip. Methods.

[10] Moran S, Vizoso M, Martinez-Cardus A, Gomez A, Matias-Guiu X, Chiavenna SM, Fernandez AG, Esteller M (2014). Validation of DNA methylation profiling in formalin-fixed paraffin-embedded samples using the Infinium HumanMethylation450 Microarray. Epigenetics.

[11] Siegel EM, Berglund AE, Riggs BM, Eschrich SA, Putney RM, Ajidahun AO, Coppola D, Shibata D (2014). Expanding epigenomics to archived FFPE tissues: an evaluation of DNA repair methodologies. Cancer Epidemiol Biomarkers Prev.

[12] Pidsley R, Zotenko E, Peters TJ, Lawrence MG, Risbridger GP, Molloy P, Van Djik S, Muhlhausler B, Stirzaker C, Clark SJ (2016). Critical evaluation of the Illumina MethylationEPIC BeadChip microarray for whole-genome DNA methylation profiling. Genome Biol.

[13] de Ruijter TC, de Hoon JP, Slaats J, de Vries B, Janssen MJ, van Wezel T, Aarts MJ, van Engeland M, Tjan-Heijnen VC, Van Neste L (2015). Formalin-fixed, paraffin-embedded (FFPE) tissue epigenomics using Infinium HumanMethylation450 BeadChip assays. Lab Invest.

[14] Olsson M, Beck S, Kogner P, Martinsson T, Caren H (2016). Genome-wide methylation profiling identifies novel methylated genes in neuroblastoma tumors. Epigenetics.

[15] Aryee MJ, Jaffe AE, Corrada-Bravo H, Ladd-Acosta C, Feinberg AP, Hansen KD, Irizarry RA (2014). Minfi: a flexible and comprehensive Bioconductor package for the analysis of Infinium DNA methylation microarrays. Bioinformatics.

[16] Fortin J-F, Triche T, Hansen K. Preprocessing, normalization and integration of the Illumina HumanMethylationEPIC array with minfi. Bioinformatics. 2017;33(4):558-60. 10.1093/bioinformatics/btw691 PMC5408810 28035024

[17] Triche TJ, Weisenberger DJ, Van Den Berg D, Laird PW, Siegmund KD (2013). Low-level processing of Illumina Infinium DNA Methylation BeadArrays. Nucleic Acids Res.

[18] Hovestadt V, Zapatka M: conumee: Enhanced copy-number variation analysis using Illumina 450k methylation arrays., R package version 0.99.4. In*.* URL: http://www.bioconductor.org/packages/release/bioc/html/conumee.html; 2015. Accessed 1 Dec 2016.

